# Strategies that enabled access to chronic care during the COVID-19 pandemic and beyond in South Africa

**DOI:** 10.4102/hsag.v29i0.2412

**Published:** 2024-03-29

**Authors:** Sheillah H. Mboweni

**Affiliations:** 1 Department of Health Studies, Collage of Human Sciences, University of South Africa, Pretoria, South Africa

**Keywords:** chronic care, enabling strategies, patients with chronic diseases, treatment, access to care

## Abstract

**Background:**

The COVID-19 epidemic has revealed disturbing information about how chronic diseases are treated globally. Healthcare providers and coronavirus response teams have primarily reported on how individuals with chronic conditions sought care and treatment. However, individuals’ experiences of patients are yet unknown.

**Aim:**

This study aimed to explore those strategies that enabled patients with chronic diseases access to chronic care and treatment during and beyond the COVID-19 pandemic.

**Setting:**

The study was conducted in the predominantly rural district of the Northwest Province, South Africa.

**Methods:**

An explorative qualitative research design was followed. Information-rich participants were chosen using a purposive sampling technique. Individual face-to-face interviews were used to gather data. Data saturation was achieved after interviewing *n* = 28 people in total. The six steps of Braun and Clarke thematic data analysis were used to analyse the data.

**Results:**

The study revealed three themes, which includes improved healthcare structural systems, shift from traditional chronic care to digital care services and medication refill and buddy system.

**Conclusion:**

The findings of this study revealed a range of effective and noteworthy approaches that facilitated access to treatment and continuity of care. As a result, enhancing telemedicine as well as structural systems such as appointment scheduling, decanting choices, mobile and medication home delivery can improve access to care and treatment.

**Contribution:**

The burden of disease and avoidable death will be eventually addressed by maximising the use of telemedicine and sustaining the new norm of ongoing care through digital and remote care and decanting strategies.

## Introduction

Unsettling truths concerning the treatment of chronic diseases around the world have come to light as a result of the COVID-19 pandemic (Fekadu et al. 2022). According to Labrique et al. (2018), there is a need for a global shift from traditional approaches to digital health because it appears that the health system is ill-equipped to maximise the health of people with chronic diseases (PWCDs). Moreover, people from marginalised populations and low- and middle-income countries (LMICs) are highly affected by chronic diseases such as non-communicable diseases (NCDs) and human immunodeficiency virus (HIV), worsened by poor access to technology and have a negative impact on the provision of digital remote chronic care (WHO 2022). Additionally, according to World Health Organization (WHO) (2021a), LMICs accounted for 77% of all NCD deaths globally each year by 2021, with 15 million of those deaths being premature deaths occurring in people between the ages of 30 and 69 years. The leading cause of death from NCDs are cardiovascular diseases, followed by cancer, chronic respiratory diseases and diabetes mellitus (WHO 2021a). In addition to its negative effects on health, chronic diseases also has economic and emotional consequences (Cockerham, Hamby & Oates 2017).

World Health Organization (2018) research states that by boosting expenditures in the prevention and treatment of chronic diseases like heart disease and cancer, which adds up to US$1.27 per person per year in costs, the world’s poorest countries may gain US$350 billion by 2030. Such measures would prevent the loss of more than 8 million lives within the same period. Therefore, it is essential to enhance patient access to screening, detection, treatment and chronic care during and beyond medical emergencies.

The COVID-19 pandemic disrupted plans and progress for chronic disease management. According to Chudasama et al. (2020), NCDs including people living with HIV (PLHIV) on antiretroviral treatment (ART) were the most impacted by the reduced access to routine care, in addition to being more susceptible to complications and death from COVID-19 (Fekadu et al. 2021). It becomes imperative to strike a compromise between protecting PWCDs from COVID-19 and ensuring that they continue to participate in their disease management and that they receive seamless chronic care in a safe manner (Hacker et al. 2021). Although it may appear that healthcare use concerns are getting better overall, neither patients nor healthcare providers feel this way. To address long-term COVID-19 sequelae, including re-establishing trust between the healthcare system and PWCDs, it is crucial to raise awareness and collaborate on solutions and innovations that will enhance access (Hacker et al. 2021). According to De Maria et al. (2021), patients with chronic illnesses reported varying levels of self-care during the early months of the COVID-19 pandemic. The study further indicated that some patients with chronic illnesses intensified their self-care behaviours while others neglected it. Danhieux, Buffel and Pairon (2020), in a study in Belgium, documented changes in risk classification, self-management support, and healthcare organisation, which led to a drop in chronic care activities resulting in fewer consultations, worsened by staff members responsible for chronic care infected by the COVID-19. Consequently, it is critical to identify the strategies that enabled PWCDs to access chronic care and treatment both during and after the pandemic’s hard lockdown periods, based on their individual experiences. Furthermore, this permits the researcher to provide policymakers with evidence-based strategies that will ensure continued access to care and treatment.

According to Brzoska, Erdsiek and Waury (2017) and Jahangir, Irazola and Rubinstein (2012), enabling strategies refer to the practical aspects of receiving care through social support systems such as family, friends and neighbours as well as structural resources found in health facilities that can help or increase the likelihood that services will meet the patient’s medical needs. Income, health insurance, the availability and accessibility of medical services, wait times or regular sources of care and transportation are also aspects that may influence access to chronic care (Brzoska et al. 2017).

A systematic literature review study by Mboweni and Risenga (2022) on the impact of COVID-19 on the management of chronic disease argued that most studies were based on the opinions, viewpoints and perspectives of the researchers, and they stressed the importance of conducting empirical studies to examine the lived experiences of PWCDs. This will provide a better insight into how they managed to access chronic care and treatment. Similarly, other researchers (Madhi & Nel 2021; Aimrane et al. 2022) ascertain that most international studies concentrated on the epidemiology and scope of COVID-19 rather than the experiences of PWCDs. Tiotiu et al. (2021) affirm that few practitioners had a systematic strategy and indicated worries about patients who were already poorly controlled, and there was no transition to digital support that could detect and contact high-risk patients for early follow-up. In addition, Stamm et al. (2022) indicate that previous healthcare disparities for underprivileged people worsen during difficult times. Between 5.6% to 14.6% of participants in a study in Asia experienced worsening NCDs and socioeconomic crises along with decreased access to care (Singh, Kaushik & Johnson 2021). Again, the perception and access to chronic care during COVID-19 differed widely between cultures and countries, and it is imperative to explore strategies used in a South African context (Nwagbara et al. 2021).

Despite already having problems with the integrated chronic disease management (ICDM) approach, which was developed in response to the twin burden of HIV and/or acquired immune deficiency syndrome (AIDS) and NCDs, South Africa was badly disrupted by the pandemic and while PLHIV were prioritised, those with NCDs were neglected (Peer et al. 2020). Similarly, Ameh et al. (2017) argued that such health facilities struggle with a lack of equipment and supplies, drug stockouts, tight appointment schedules, ineffective defaulter tracing and lengthy wait times. This raises concern and calls for an urgent need for innovative strategies to fastrack access to treatment and care as well as to limit exposure and fear of contracting COVID-19. According to Adams et al. (2021), the majority of PWCDs, particularly those over 45 years who lived within one kilometre of the clinic, expressed interest in pharmaceutical delivery service rather than spending more than 6 h waiting at a facility. Mboweni and Risenga (2023) conducted a study where PWCDs identified barriers to accessing chronic care services, such as long waits, staff shortages and negative staff attitudes. People with chronic diseases emphasised the need for improved access, prompting the researcher to conduct this study to explore strategies that will enhance access to chronic care and treatment services not only during COVID-19 but beyond.

## Purpose of the study

The purpose of this study was to investigate and describe strategies that enabled PWCDs access to chronic care and treatment services, which will be documented as lessons learned from the COVID-19 pandemic and beyond based on PWCDs lived experiences to enhance continuity of care and for use in future pandemics.

## Research methods and design

The strategies that aided access to chronic care and treatment for PWCDs during the COVID-19 pandemic and beyond the primary healthcare (PHC) settings were obtained using an exploratory descriptive qualitative research approach. This problem-based approach seeks to gain a deeper understanding of how to explore, interpret, and describe the phenomenon within the PHC context. The combination of exploratory and descriptive elements provided a rich, detailed insight, viewpoints, perspectives and experiences as lived by PWCDs during their struggle to access treatment and care (Brink, van der Walt & Van Rensburg 2018; Polit & Beck 2017:156). Given that PWCDs experienced these strategies in a particular environment, this was thought to be the ideal way to explore and describe those approaches to generate evidence-based knowledge and practices (Gray, Grove & Sutherland 2018; Polit & Beck 2017).

## Study setting

The study was conducted in selected public PHC facilities in the Bojanala district, the largest district in Northwest Province. The district is primarily rural and is densely populated with informal settlements, farming and mining areas as well as a high proportion of migrant workers. Ajaero et al. (2021) state that there is a high correlation between areas with a high migrant population with increased prevalence of HIV and NCDs, and this is related to health disparities.

### Population and sampling

Non-probability purposive sampling was used to identify and select information-rich participants followed by convenience sampling of all participants who met the inclusion criteria and attended the chronic disease clinics. Participants were PWCDs, including NCDs and PLHIV, 18 years and above, and willing to share their first-hand experiences and perspectives during and after the COVID-19 pandemic lockdown period. Of the 50 PWDCs who were contacted, only 28 accepted to participate in the study. With this sample (*n* = 28), data saturation was attained, which happens when new information from more participants runs out and emergent themes begin to resemble one another (Gray et al. 2018; Polit & Beck 2017).

### Data collection

Individual face-to-face unstructured interviews were conducted by the researcher, who has a Ph.D. in Nursing with experience in research, education, training and practice in the management of communicable and NCDs at PHC level and is a senior lecturer from an open distance electronic learning (ODeL) institution. Except for the therapeutic nurse-patient contact, there was no particular connection between the researcher and the patients. After receiving approval from the district management team, operational managers and professional nurses (PNs) were used as gatekeepers. Using the participant information (PIL), PNs informed PWCDs about the study during health education sessions. This was distributed to the facilities by the researcher via email to the operational managers to share with PNs managing chronic diseases. The PIL thoroughly described in detail the study’s goals, advantages and risks. Those interested were referred to the researcher. The researcher contacted them and those who agreed to participate were interviewed.

Individual interviews with participants were done in a private room with no interruptions once informed written or verbal agreement from those who could not read or write was acquired. Participants were allowed to share their thoughts, feelings, and experiences freely. Although there was no compensation or incentive for taking part in the study, participants were provided water and fruits since they had a chronic illness and had to wait a long time to receive care and participate in the study. The interview guide was used and first piloted in two PHC facilities. This was an opportunity to refine the questions and questioning skills. These facilities and results were excluded from the main study. Three different data-gathering approaches were used by the researcher throughout the data collection phase, which is the use of audiotape, fieldnotes and observation of participants’ feelings and reactions to questions asked (Polit & Beck 2017). The data collection phase took place between April and June 2022. The interviews were recorded on audio tape with permission from participants. Patients were questioned in Setswana, and because the researcher has lived and worked in the province for 8 years and speaks it fluently, their comments were translated into English during transcription. Participants were inquired as to the following: Please can you share your experience of accessing chronic care and treatment during the COVID-19 epidemic lockdown? There was more probing after that. The duration of each interview was between 30 and 45 min. In addition to participant demographics, facility characteristics such as operational hours, decanting and use of telemedicine were captured. Nonverbal answers observed, including the participants’ emotions, were noted in field notes. Participation was optional, and some people said they were rushing, while others said they were hungry because they had arrived as early as 6 a.m. and were exhausted. The majority of PWCDs viewed the interview session as a debriefing opportunity to share their own narratives and first-hand accounts of the COVID-19 pandemic lockdown.

Infection prevention and control (IPC) rules and policies for the COVID-19 pandemic health centre and the university were adhered to by making sure the area was well aired, hand sanitiser and masks were accessible, and there was enough space to maintain a distance of 1.5 m.

### Data analysis

Data analysis was conducted simultaneously with data collection; this is the norm in case of qualitative studies (Creswell & Creswell 2018). Table 1 summarises the participant and health facility variables that were recorded and analysed using descriptive statistics. A reflective notebook was kept, and entries about potential role conflicts and areas of interest were recorded in it. Gatekeepers were employed to guarantee impartiality. The researcher tried to put aside any prior beliefs regarding how COVID-19 affects the management of patients with chronic diseases to retain objectivity. Without using any software, the following six steps of Braun and Clarke’s (2021) thematic analysis were used: The researcher: (1) transcribed the data verbatim, carefully read and reread it until the researcher became familiar with the data in order to gain sense and meaning while making notes. At this point, (2) similar data were identified and different codes were generated, (3) similar coded data were further sorted using mind mapping in a way that showed relationships into themes and sub-themes, (4) the transcribed data and developing themes were further reviewed by the researcher and also presented to a more seasoned independent researcher in qualitative methods to critically review the process and to confirm quality. Feedback was discussed, two interviews were repeated for validation and member checks were done. This led to adjustments and changes of theme names, (5) The themes were named and described appropriately in this stage to address the research question and three themes emerged from the study findings. (6) Exemplars were located in the form of quotations to add texture to themes and sub-themes when writing the report of the study As some material was unclear, two interviews were repeated, and revisions were made after that. Additionally, the experienced researcher received a draft of the publication to review, examine the consistency of the data and conclusions and offer constructive criticism (Creswell & Creswell 2018; Gray et al. 2018).

**TABLE 1:** Demographic characteristics of the participants and health facilities.

Demographics	Frequency	%
**Gender**		
Female	21	75.00
Male	7	25.00
Total	28	100.00
**Age in years**		
18–28	4	14.28
29–39	6	21.42
40–50	12	42.85
50–60	5	17.85
60 and above	2	7.14
Total	28	100.00
**Residential area**		
Rural	22	78.57
Urban	4	14.28
Semi-urban	2	7.14
Total	28	100.00
**Type of chronic disease**		
Hyperparathyroidism (HPT)	7	25.00
Diabetes mellitus (DM)	3	10.71
Asthma	4	14.28
HIV on ART	10	35.7
With comorbidity	4	14.28
Total	28	100.00
**Source of income**		
Employed	5	17.85
Government grant	4	14.28
Self-employed (selling)	2	7.14
Unemployed	17	60.71
Total	28	100.00

HIV, human immunodeficiency virus; ART, antiretroviral treatment.

### Ethical considerations

As the research involves human participation, the Nuremberg code of ethical code was adhered to in order to protect the rights of all participants and institutions involved. The study was approved by University of South Africa (UNISA) ethics committee under Ref number 90476050_CREC_CHS_2021 for the period 29 October 2021–2024. Permission to conduct the study was obtained from the Northwest Province Department of Health (ref. NW_202201_004).

Numbers were utilised instead of participant’s names and no personally identifiable information was used to adhere to ethical standards and the *Protection of Personal Information (POPI) Act*, which is in place in South Africa starting from April 2020. Confidentiality and anonymity were also preserved through this process. The consent forms and recorded audio tapes were stored apart and secured, and field notes were saved electronically and password protected. Data security was improved by keeping it on a laptop with password-protected access available only to the researcher. The study’s participants gave their consent voluntarily, both written and verbal consent were obtained as some were unable to write, and they were made aware that the interview would be audio recorded and that they could opt out at any point without incurring any costs. Debriefing was done after each interview, participants were asked how they felt after the interview. Those who experienced emotional discomforts were reassured and calmed down as some questions reminded them of the misery brought by the COVID-19 pandemic. Some participants were referred to social workers and case facilitators based in the facility for professional counselling and support. However, most felt like they were in a debriefing session where they managed to voice out their views and feelings. No adverse events were experienced or observed during the interview.

### Trustworthiness in qualitative research

In qualitative research, trustworthiness is a method of ensuring data quality or rigour (Brink et al. 2018). The credibility, dependability, confirmability and transferability of the study were affirmed by several strategies aimed at increasing trustworthiness and obtaining participants’ lived experiences. In line with Lincoln and Guba 1985 as cited in Creswell and Creswell (2018), trustworthiness in this study was enhanced as follows: Implementing techniques to boost credibility, such as spending more time with participants about 30–45 min. Carrying out a member check during which the researcher went back to participants to ensure that their experiences were appropriately recorded improved dependability. Collecting in-depth lived experiences of PWCDs applying explorative descriptive design using interviews from the selected facilities until data saturation was reached increased transferability. This also allowed the researcher to obtain a clearer insight into strategies used to access chronic care during and beyond the COVID-19 pandemic. In a location other than where the study took place, a preliminary investigation was conducted. The researcher also used reflexibility to increase credibility. Reflexivity is self-awareness on the part of the researcher, used to avoid bias in assessments and raise the standard of the study. Conformability was enhanced by keeping audit trails of audio-recorded interviews and careful documentation of field notes and making this available to an experienced researcher for scrutiny (Creswell & Creswell 2018; Gray et al. 2018; Polit & Beck 2017).

## Results

As indicated in Table 2, the study revealed three themes that include: (1) Structural strategies that enabled access to health services for PWCDs; (2) shift from traditional chronic care to digital care services and (3) medication collection and refill through Buddy system. The sociodemographic characteristics of participants that have influenced the study findings are summarised in Table 1.

**TABLE 2:** Themes and sub-themes that emerged from the study findings.

Theme		Sub-theme
1.	Structural strategies that enable access to chronic care and treatment services by PWCDs	1.1. Compliance with COVID-19 health regulations 1.2. Prioritisation of high-risk individuals 1.3. Use of knowledge about chronic diseases 1.4. Implementation of decanting strategy 1.5. Use of mobile clinics and home delivery 1.6. Use of appointment system
2.	Technological and digital strategies that enable access to chronic care and treatment	2.1. Telemedicine and/or mHealth remote care – SMS reminders, telephone counselling services2.2. Remote care to enhance continuity of patient centred care from health facility.
3.	Medication collection and refill strategies that enhance treatment	3.1. Buddy system medication collection

PWCDs, people with chronic diseases; COVID-19, coronavirus disease 2019, SMS, short message service.

### Socio-demographic characteristics of the study participants

Table 1 reflects the demographics of both the facility and the participants involved in the study. A total of 28 patients from the four selected facilities were interviewed, and data saturation was reached with this sample (Mboweni & Risenga 2023). The demographic data reflected the socioeconomic status of the participants and how this situation had influenced access to chronic care, self-care and treatment during the COVID-19 pandemic, more especially during the hard lockdown. Most of the participants (75%) were females, and the remaining 25% were males. In terms of age, most participants were between the ages of 40 and 50 years at 42.85% with the lowest percentage (7.14%) being 60 years old and above. Most participants were PLHIV on ART (35.7%), the next largest group comprised those with hypertension (HPT) (25.0%); the smallest number were participants with cardiac conditions and the lowest number was participants with comorbidities (14.28%). Most of the participants (78.57%) were from rural areas, (Mboweni & Risenga 2023).

Most (60.71%) of the participants were unemployed: 17.5% worked for an employer and 7.14% were self-employed. Sources of income were government grants (14.3%), employers (17.9%) and selling (7.1%) (Mboweni & Risenga 2023). Most of the study participants were of low socioeconomic status and depended on public health facilities for chronic care and treatment services.

## Themes: Strategies that enabled access to chronic care and treatment services by people with chronic diseases

### Theme 1: Structural strategies that enable access to chronic care and treatment services

Participants stated that they were able to access chronic care and remain on treatment by following COVID-19 health regulations when visiting the facility; utilising acquired knowledge of chronic diseases at home; accepting the decanting strategies offered; adhering to the appointment system aimed at reducing overcrowding in health facilities even though they still experienced long waiting hours and utilisation of mobile clinics, even though they were disrupted at some point.

#### Compliance with COVID-19 health regulations

Participants revealed that at the beginning of the pandemic, most patients did not want to comply with such regulations as wearing of masks, maintaining a social distance of 1.5 m and sanitising as it caused confusion and delayed care. Participants reported that:

‘Nurses always teach us to stay at home to prevent infection and complications related to comorbidity.’ (P1, Female, 42 years)

‘I always wear my mask properly, close mouth and nose, it was not simple at first but we managed to comply.’ (P2, Male, 45 years)

#### Prioritisation of high-risk individuals

Participants reported that health facilities tried to fast-track and prioritise people with chronic diseases were at greater risk of COVID-19 infection; however, they were many and with the shortage of staff they will have to wait longer sometimes. Participants reported that:

‘Nurses tried to first see us, it was good but some days she was just alone and did not work for us days.’ (P8, Male, 58 years)

‘I did not spend much at the clinic, they see us first.’ (P17, Female, 26 years)

#### Use of knowledge about chronic disease

Participants applauded nurses for investing in health education during consultations as it helped patients cope with their conditions during the lockdown when they were alone without family and community-based organisation (CBO) support. Participants stated that:

‘Knowledge is power. I remembered all that I have been taught and implemented it, including exercising, healthy diet and taking treatment as prescribed.’ (P4, Female, 49 years)

‘I started calculating dates for going to the clinic for Viral load blood monitoring and I go back to the clinic.’ (P3, Female, 51 years)

#### Implementation of decanting strategies

Participants indicated that they were offered to choose from the free-of-charge innovative decanting strategy of repeat prescription collection services (RPCS). This was an initiative by the Department of Health Central Chronic Medicines Dispensing and Distribution (CCMDD) programme so that PWCDs no longer need to come to the facility and experience long waiting hours, which increased their risk of infection. People with chronic diseases could choose from services such as Spaced Fast Lane (SFL), where they collect treatment in a designated area within the health facility or an external pick-up point in approved areas such as local pharmacies, general practitioners, shops or CBOs near their homes or workplaces as well as community adherence clubs. However, the adherence clubs were disbanded. Participants reported that:

‘We were so happy to be decanted in external pick up, where we collect medications at local supermarket or pharmacy as we longer wait long at the clinic.’ (P 21, Female, 50 years)

‘The clinic was so relieved of overcrowding, and getting COVID-19 infection as most of the patients were just given a space where we just come in and collect and go home.’ (P22, Male, 42 years)

‘I knew about decanting before COVID-19 and I refused, but because of fear I came to request to be decanted on my own, seeing the benefits.’ (P15, Male, 50 years)

They also indicated that they were offered the option to collect their treatment outside the facility structure or go to SFL. This was appreciated as they now had access to medication through the Pelo box without having to go inside the facility.

One participant showed appreciation with a smile saying:

‘I just receive an SMS with a code in my phone then go to the Pele box that is outside the facility and get my treatment.’ (P7, Male, 23 years)

Another participant reported that:

‘I chose the Spaced Fast Lane, where I skip the queue and just go to office where we just pick up treatment.’ (P9, Female, 36 years)

#### Use of mobile clinics and home delivery

The study revealed that PWCDs staying far from the facility were served by mobile clinics that provided PHC services, including chronic care and treatment, and some came to deliver treatment at the doorsteps. The participants therefore appreciated the initiative and availability of mobile clinics even though it was sometimes interrupted and suggested that this was something that should be continued and sustained to improve access to health services. Participants expressed their concern by saying:

‘We had a mobile visiting our farm during lockdown but it is not reliable as it is no longer coming, I wish they can continue doing so.’ (P13, Female, 45 years)

‘I stay far from the clinic, there is no transport and traveling was restricted during level 5–4 lockdown, oooh, it was so helpful that the mobile came and home delivery was excellent.’ (P8, Male, 58 years)

#### Use of appointment system

Participants explained that the health facilities implemented an appointment system whereby small groups or cohorts were given dates to visit the health facility for chronic care and treatment services. While this aimed to reduce overcrowding, and long waiting hours during COVID-19, it was suggested that this should be sustained and implemented beyond COVID-19 as the new normal in PHC facilities. One participant was excited while stating that:

‘Coming in the clinic in small groups is great and by an appointment reduces overcrowding and long waiting hours.’ (P12, Female, 46 years)

Another participant smiled and was recorded saying:

‘Why can’t they offer everybody appointments so that we do not queue, including those we come for immunisation, this is good.’ (P24, Female, 47 years)

### Theme 2: Technological and digital strategies that enable access to chronic care and treatment

The study revealed that there has been a shift from traditional chronic care to digital remote care services through the use of technology in healthcare facilities. Staff now call to provide counselling and remind them of appointment dates using telemedicine and/or mHealth. The use of telephone, cell phone and other wireless technology in medical care has allowed PWCDs to report side effects and other psychological effects related to their conditions.

#### Telemedicine and/or mHealth

Participants expressed their pleasure about changes brought about by the pandemic. They now receive in the comfort of their own home, counselling and support through telephone and SMS appointment reminders and notifications that they are due for blood assessments. The healthcare system has strengthened the use of mHealth to educate their patients and increase surveillance of COVID-19 and other epidemics such as HIV and/or AIDS and tuberculosis (TB). Participants happily stated:

‘It’s nice to receive a phone call from counsellors and check on us.’ (P19, Male, 55 years)

‘I’m always reminded of my appointment through SMS or a call.’ (P17, Female, 26 years)

Participants also indicated that there is improvement in reducing long waiting hours:

‘We go to the clinic in small groups according to our appointment and we are no longer going through the long queues, even though sometimes they make us wait.’ (P16, Female, 57 years)

#### Remote care to enhance continuity of patient-centred care

The study showed that PWCDs received overwhelming support and remote care from health facilities to enhance the continuity of patient-centred care. Patients are unique and need individualised care. The PHC introduced case facilitators or managers from the United States President’s Emergency Plan for AIDS Relief (PEPFAR) funded partners in the district. A case manager sometimes called a case facilitator as a psychosocial professional who is assigned a certain number of PWCDs in PHC facilities to apply their adherence counselling skills, coordinate and manage the care of such clients in collaboration with other multidisciplinary teams by assessing the needs, developing a care plan, evaluating its effectiveness and terminating it if no longer needed or helpful. These facilitators can be social workers or those with degrees in social sciences and psychology (Giardino & De Jesus 2023). They used phones and social media to provide remote care such as health education, information adherence counselling, self-care intervention, support, as well as coping strategies and lifestyle modification. They also send SMS reminders for treatment and clinical review appointments; all of which have also contributed positively to the mental health of the patients. They also receive calls should they miss treatment. As evidenced by participant quotes, most PWCDs’ mental health and adherence to treatment improved.

Participants reported a feeling of acknowledgment stating that:

‘The introduction of case facilitators or case managers brought a relief as we were not having that in our clinic and assist us to get information, health education to cope, live with the disease, disclose our conditions to family members for support.’ (P10, Female, 61 years)

‘I managed to deal with my mental health issues after being diagnosed with hypertension while having HIV, my fear and stress were relieved through telephone counselling and I can take both treatments.’ (P16, Female, 57 years)

‘During each telephone call or visit counsellors and clinic staff stress the importance of healthy lifestyle or behaviour to us so that they boost their immune system.’ (P20, Female, 38 years)

### Theme 3: Medication collection and refill strategy through the Buddy system

The study revealed that the healthcare system supported PWCDs by allowing their buddy, which can be close friends, neighbours, colleagues or family to come and collect their medication to ensure uninterrupted access to medication during the pandemic, and this continues beyond. This played a major role in adherence and prevention of treatment interruption. They were expected to register this person as a buddy at the clinic so that he and/or she could be allowed to collect treatment.

Support from family members and friends was considered important by most PWCDs during the pandemic lockdown period as many lived in fear of contracting the disease, not knowing how to access treatment and care and how to keep themselves healthy. Participants revealed with a smile that:

‘My family supported me a lot, it relieves my stress and they sometimes go to the clinic to collect my treatment.’ (P18, Female, 60 years and P5, Female, 30 years)

‘My friend and neighbours make sure that they do not come near me as a preventive strategy, but they prepare a healthy diet for me and deliver at my door as I have to take food before taking treatment.’ (P27, Female, 61 years)

## Discussions

The study findings will be discussed under the following themes: structural strategies that enabled access to chronic care services by PWCDs; shift from traditional chronic care to digital care services and medication collection and refill through the Buddy system (Kendzerska et al. 2021).

### Structural strategies that enabled access to chronic care services by persons with chronic diseases

It is evident from the study findings that compliance with health regulation during the pandemic, even though difficult, had positive outcomes as it prevented and controlled the spread of diseases and complications related to comorbidity, especially among older persons with chronic disease. This is supported by a study conducted by Jaureguizar et al. (2021), who established that the older age group had higher compliance with health regulations than the young and adolescents. This prompted the health system to adjust its chronic care model. This study makes it evident that investing in different strategies that enable access to care and treatment is beneficial to reducing the burden of diseases and achieving Universal Health Coverage (UHC) and Sustainable Development Goals (SDGs). This is supported by a study conducted in Texas by Paterick et al. (2017), which emphasised that the healthcare system and the healthcare professionals (HCPs) should devise robust strategies to prioritise PWCDs and establish a strong partnership with patients so that they are responsive to personal care and reduces hospitalisation costs

It is evident from the study findings that the use of mobile clinics increases access to chronic care and treatment. This is supported by a study conducted by Bertoncello et al. (2020), that mobile health clinics are effective in attracting hard-to-reach age groups and facilitate achieving access to care, equity, UHC and SDGs by 2030, especially in rural and disadvantaged communities. The study findings showed that effective use of the appointment system has the potential to reduce long waiting hours and overcrowding in health facilities, increasing access and thus improving patient satisfaction levels despite its challenges This is supported by a study conducted by Mozes, Mossinson, Schilder, Dvir, Baron-Epel and Heymann (2022) that PWCDs prefer an appointment system and telemedicine; however, they need a more efficient system to manage it through assessment of patient perspectives and experiences. A study conducted by Mukumbang, Orth and Van Wyk (2019) on ART treatment adherence revealed that an appointment system can be better implemented with a decanting strategy, especially in PLHIV and NCDs, and highly recommended as it improved access, adherence and retention in care. Decanting refers to the process of transferring stable PWCDs to collect their treatment outside the facility such as local pharmacies, supermarkets, post offices or use of a Pudo box free of charge or facility-spaced fast lane through the Central Chronic Dispensing and Distribution (CCMDD). This strategy is also known as ‘Dablapmeds’ in South Africa and in neighbouring countries (South Africa 2020). It is mostly funded by the Ministry Department of Health. This was started in 2014 in line with National Health Insurance (NHI) in pilot districts in the Republic of South Africa (South Africa 2020). It had over 1.9 million people before the COVID-19 pandemic and has now grown to over 4 million users to access chronic treatment at their convenient time and relieve significant pressure off the PHC facilities (Molelekwa 2021).

This was supported by a systematic literature study conducted by Tsikada (2020) among chronic psychiatric patients that the appointment system can improve access and adherence to medication. In addition, Subramaniam Kalianan et al. (2021) in a study on the Malaysian public healthcare system affirm that patients prefer flexible scheduled appointments such as after-hours, weekends and evenings to access chronic care services. A study conducted in South Africa by Egbujie et al. (2018) supports the fact that effective implementation of an ideal clinic model and decanting strategy in health facilities can change patient waiting times.

### Shift from traditional chronic care to digital care services

It is apparent from study findings that a strong PHC system in rural communities has shifted from the traditional methods of facility chronic care to digital technology and remote care services. This is needed to make a significant contribution to a successful health system response. This was supported by Stamm et al. (2022), who stated that disadvantaged individuals suffered a level of healthcare disparity never seen before because of a lack of technology to implement telemedicine. Coronavirus disease 2019 stigma varies throughout cultures and countries, especially in the African region with some patients rejecting telehealth when offered (Gcabashe & Matambo 2020). According to Hu (2018) and Mitchell and Kan (2019), leveraging digital tools and systems for e-health and telemedicine is a healthcare investment and can be carried out through the development of a legal and legislative framework, guidelines and regulations. This was supported by Shah, Alkureishi and Lee (2021); the US Japan Council business report (2020) and the American Hospital Association (2019) that telemedicine or telehealth is the best way in which patients can receive care from the HCPs during medical emergencies and beyond. This can happen through phone calls, emails, online portals, my charts, remote patient monitoring, video calls and recording health history through a secure electronic communication system to HCPs. This enhances interactions and improves patient care as well as capacity development regarding self-care quality. Digital tools help maintain continuity of care for PWCDs and provide remote patient-centred and flexible care (Granström et al. 2020).

According to Wong et al. (2021), the potential clinical practice benefits of a virtual-care framework during a pandemic include more effective routine disease monitoring, improved patient satisfaction and increased treatment compliance and follow-up rates, which can be sustained for continuous use (Kaur, Chauhan & Mascarenhas 2023). This was supported by Stamenova et al. (2022) in their study, which revealed that PWCDs have shown an upsurge in the usage of virtual care. At the beginning of the pandemic, laboratory tests and hospitalisations decreased significantly for both low and high-virtual-care usage groups. High users’ hospitalisation rates increased, and the decrease in in-person care during the pandemic was accompanied by an increase in virtual care (Durant et al. 2020). This was supported by Kendzerska et al. (2021) who showed that during the COVID-19 pandemic, in-person care for individuals with chronic conditions decreased because of government restrictions on elective and non-urgent healthcare visits. The study further affirms that there were higher utilisation rates of telemedicine compared to the pre-COVID-19 period (Kaur et al. 2023). However, more needs to be done to ensure timely and effective access to telemedicine, particularly for individuals with lower digital literacy and warrants further investigation (Kaur et al. 2023; Kendzerska et al. 2021). A study conducted in Germany by Reitzle et al. (2021) revealed that the majority of participants’ access to medical care was ensured during the COVID-19 pandemic through technological advancement. Telemedicine complemented the access to medical appointments. Contrarily, Singh et al. (2021) argued that PWCDs faced difficulties in accessing healthcare as few have access to teleconsultations, especially in disadvantaged communities. Most participants reported adverse economic impact by the pandemic, which led to interrupted use of technological devices as they cannot afford (Monte 2021).

Remote care that included adherence counselling and access to information about their condition including COVID-19 was provided by case facilitators. These was also useful linking and referring of PWCDs to other members of the multidisciplinary team telephonically. This support cannot be overlooked as it enabled PWCDs to adhere to treatment. This was supported by a study conducted by Akyirem et al. (2022), which stressed that education about self-care should form part of remote interventions and communication with patients, family and friends. The study further emphasised that remote counselling and support enhance acceptance of the disease and is necessary to instil hope and a sense of purpose in the lives of PWCDs. Conversely, while it is necessary to educate PWCDs, HCPs should empower PWCDs to fight for their rights, deal with stigma and discrimination and foster respect and dignity. Another study by Kemp, Mead and Fisher (2022) highlighted that strengthening and maintaining psychosocial support services remotely enhances treatment adherence. Additionally, Rahia (2019) indicated that family-based interventions are not only effective in accessing treatment but help to reduce anxiety and depression in PWCDs. In the heat of a crisis, the voice of the patient needs to be heard. Therefore, remote patient engagement should go beyond self-care and impact healthcare delivery policies and patient centred care based on patient experiences (Krist et al. 2017). Hanrahan et al. (2019) argued that patient-centred care and retention to care strategies should be strengthened to optimise chronic care. A study by Tshuma et al. (2017) in South Africa showed external treatment pick-up point enhances access and adherence to treatment while also addressing stigma and discrimination.

### Medication collection and refill services through the Buddy system

It is evident from study findings that apart from telemedicine, those who cannot afford it opted for a Buddy system. This was a good strategy that enabled PWCDs from disadvantaged communities to access treatment using their close friends, neighbours, family and colleagues. Most PWCDs were afraid to contract COVID-19 infections in health facilities and transport facilities and felt relieved because of this option. This was supported by a systematic review study conducted by Wiig et al. (2020), which revealed that family members can collect treatment; however, this should be based on country health regulations and policies. Family and friends played a major role in collecting medication for PWCDs and also bringing them to the facility for clinical review. In South Africa, PWCDs who are in stable condition are given a choice to collect treatment outside the facility or can send a buddy to collect treatment as long as it is not time for clinical review or collection of specimens. However, they are expected to register these buddies in the facility to ensure control and confidentiality (South Africa 2020). This was supported by McCool et al. (2022) palliative care study, which found that the Buddy system fosters resilience and lowers stress in addition to facilitating access to care and treatment. In a similar vein, Schrøder et al. (2022) confirm that a Buddy system is an effective peer support initiative that may also be used as a mentoring strategy for healthcare professionals and students in clinical learning and teaching. The Buddy system can strengthen the healthcare system in many ways.

### Limitations

The study’s participants and health institutions were chosen from just one of the Northwest Province’s four districts; thus its conclusions cannot be extrapolated to other regions. Similar investigations might be carried out in other districts, though, where lockdown rules for the COVID-19 epidemic were also felt. Data were gathered after lock-down level 2, but it was not until after that that it was analysed. The results gave rise to an evidence-based strategy that was utilised to maximise the adoption of the chronic care model and ongoing access to care and treatment. Additionally, few studies focus on the actual lived experiences of PWCDs; as a result, this study offered a deeper understanding and information that is useful for the chronic care model, society and the healthcare system.

## Conclusion

The study aimed to explore the strategies that enabled access to chronic care and treatment during medical emergencies, in this case, the COVID-19 pandemic. Planning and implementing evidence-based practice based on the experiences of PWCDs requires a patient-centred approach as it gives patients a better understanding of what may and may not work. The results of the study make it clear that the health system should keep using digital health services to monitor patient outcomes remotely while enhancing and sustaining the transition from conventional methods of chronic care delivery to telemedicine. To maximise disclosure, treatment adherence and retention within care, HCPs should also encourage PWCDs to seek help from their buddies and community-based support groups. To empower both PWCDs and HCPs, personal and self-care activities should be incorporated into remote chronic care
